# Experimental Characterization of Composite-Printed Materials for the Production of Multirotor UAV Airframe Parts

**DOI:** 10.3390/ma16145060

**Published:** 2023-07-18

**Authors:** Tomislav Šančić, Marino Brčić, Denis Kotarski, Andrzej Łukaszewicz

**Affiliations:** 1 Department of Mechanical Engineering, Karlovac University of Applied Sciences, 47000 Karlovac, Croatia; tomislav.sancic@vuka.hr; 2 Department of Engineering Mechanics, Faculty of Engineering, University of Rijeka, 51000 Rijeka, Croatia; marino.brcic@riteh.hr; 3 Institute of Mechanical Engineering, Faculty of Mechanical Engineering, Bialystok University of Technology, 15-351 Bialystok, Poland

**Keywords:** multirotor UAV airframe parts, additive manufacturing, continuous fiber fabrication, fiberglass reinforcement, material experimental characterization, uniaxial tensile test

## Abstract

In this paper, the characterization of 3D-printed materials that are considered in the design of multirotor unmanned aerial vehicles (UAVs) for specialized purposes was carried out. The multirotor UAV system is briefly described, primarily from the aspect of system dynamics, considering that the airframe parts connect the UAV components, including the propulsion configuration, into a functional assembly. Three additive manufacturing (AM) technologies were discussed, and a brief overview was provided of selective laser sintering (SLS), fused deposition modeling (FDM), and continuous fiber fabrication (CFF). Using hardware and related software, 12 series of specimens were produced, which were experimentally tested utilizing a quasi-static uniaxial tensile test. The results of the experimental tests are provided graphically with stress–strain diagrams. In this work, the focus is on CFF technology and the testing of materials that will be used in the production of mechanically loaded airframe parts of multirotor UAVs. The experimentally obtained values of the maximum stresses were compared for different technologies. For the considered specimens manufactured using FDM and SLS technology, the values are up to 40 MPa, while for the considered CFF materials and range of investigated specimens, it is shown that it can be at least four times higher. By increasing the proportion of fibers, these differences increase. To be able to provide a wider comparison of CFF technology and investigated materials with aluminum alloys, the following three-point flexural and Charpy impact tests were selected that fit within this framework for experimental characterization.

## 1. Introduction

Technologies of rapid prototyping enabled a great step forward in various fields. Additive manufacturing technologies, which are widely used in biomedicine [1,2] to mechatronic systems such as unmanned aerial vehicles (UAVs), play a major role. The advantages of AM technologies come to full expression in the development sector or the production of small series. Nowadays, there are numerous types of research and applications of aircraft in various sectors, such as aerial photography [3], surveillance [4], precision agriculture [5,6], transport and logistics [7], research [8,9], and many others. Due to the possibility of vertical take-off and landing (VTOL), and therefore stationary flight and moderate-speed flight, multirotor UAVs are suitable for missions that require a high degree of system autonomy. Furthermore, the performance of the propulsion system consisting of several rotors enables greater agility and maneuverability, which consequently enables precise and complex movements. That fact places them in the category of aerial robotic systems and, in general, for specialized purposes. Major manufacturers mostly offer conventional multirotor configurations that are intended for specific applications. For example, the largest multirotor UAV manufacturer DJI has several aircraft sizes that are intended for aerial photography, from the Mini to the Inspire UAV. In addition, there is a series of Agras UAVs that are intended for smart spraying tasks. These are large series of aircraft for which the frame parts are produced by conventional technologies, such as injection molding.

The multirotor type of UAV is used in more and more specific applications, and small series or unique aircraft are often required. The development of rapid prototyping technology is correlated with the development of specialized multirotor UAVs, where the key area is additive manufacturing (AM), 3D printing. There are many studies describing UAV systems that are built using additive technologies, such as fixed-wing type of UAVs [10] or considered multirotor [11]. With today’s dynamic market demands based on shorter product life cycles that require smaller production batches, there is a need to move from traditional production systems to next-generation production systems [12]. Such systems must have high flexibility and reconfigurability in order to adapt to changes in the market, and this is exactly what is achieved with reconfigurable production systems (RMS) [13]. This is made possible by a quick response to the customer’s needs by making products according to his requirements, which achieves his satisfaction. In contrast to the mass customization paradigm [14], where customers choose a product from a multitude of offered combinations, with mass individualization, the customer himself participates in the design of the product, which is suitable for the use of AM technologies, which gives advantages from the economic side due to the creation of a unique product adapted to an individual customer [15]. Increasing demands for unique customer-oriented products switch to the mass individualization paradigm, where the goal is to reduce the price of products to the level of products made by the mass customization paradigm.

AM technologies play a crucial role in the fourth industrial revolution by offering the ability to surpass the limitations of traditional production systems. Due to its flexibility, agility, and speed of placing new products on the market, AM is no longer used only for the creation of prototypes but also for the serial production of functional structural parts with the required accuracy and mechanical properties. With the development of new materials and forms of workpieces that ultimately lead to an increase in product quality, AM is used more and more often in research, especially in mechatronics, where production can be roughly divided into the design phase and the production phase itself. With this common approach, parts, or 3D objects, are created with the addition of materials, using technology by adding layers on top of each other. There are different AM technologies such as fused deposition modeling (FDM) [16], stereolithography (SLA) [17], selective laser sintering (SLS) [18], polyjet technology [19], laminated object manufacturing (LOM) [20], and others. Compared to other methods, FDM technology has its advantages, such as the variety of low-cost materials, which is why it is the most commonly used method, but there are always problems related to mechanical properties and dimensional accuracy. The most commonly used conventional materials are acrylonitrile butadiene styrene (ABS), polylactic acid (PLA), and polyethylene terephthalate glycol (PETG).

Additively manufactured polymers alongside polymer composites represent anisotropic materials [21]. With regard to the considered application of the parts, it is extremely important to identify the mechanical properties concerning the material and production parameters. In the research [22], the applicability of test methods for the mechanical characterization of test specimens manufactured with FDM technology was discussed. Furthermore, the numerical and experimental study of the PLA material compression uniaxial properties is presented in the research [23]. There are many types of research and works on the topic of improving the mechanical properties of materials that are made with FDM technology. One of the directions is joining polymer with composite materials that are widely used in research and the aerospace industry, where various types of materials and different purposes are represented, such as prototyping and production of wing structures shown in the papers [24,25]. The paper [26], gives an overview of strategies such as short fiber reinforcement (SFR), continuous fiber reinforcement (CFR), powder addition reinforcement (PAR), and other methods. Further related to the production of UAV parts, in paper [27] a novel sandwich structure consisting of an ABS base laminated with carbon fiber-reinforced polymer (CFRP) layers was proposed. The main disadvantage of such materials is the time required to manufacture such parts. The solution is imposed with new 3D printers that are based on FDM technology, known as continuous fiber fabrication (CFF). In the paper [28], an experimental investigation of the additive manufacturing of continuous carbon fiber composites was carried out.

In this paper, experimental characterization was performed for 3D-printed specimens where three AM technologies were considered. The design of a multirotor type of UAV for specialized purposes is studied, and since the need for making prototypes or small series of UAVs, AM technologies are investigated for the production of airframe parts. To achieve the UAV assembly construction more efficiently integration of the process of characterizing the materials into the design process is examined. Results for the performed uniaxial tensile test are shown, and two more tests are being preliminarily investigated, which will also be integrated into the material characterization process. The goal is to enable a comprehensive comparison with aluminum alloys with high-reliability requirements such as in the automotive industry. To conduct experimental tests, 12 series of test specimens were produced using FDM, SLS, and CFF technologies. Test specimens are subjected to mechanical testing and experimental data are processed, saved, and graphically presented for each specimen. Since the emphasis in this study is on mechanically loaded airframe parts, particular attention is given to composite materials with reinforcement for enhancing the structural integrity of the airframe. The results for different reinforcement parameters are shown, and from the aspect of maximum stress, composite specimens have up to four times higher maximum stresses in the considered parameter range compared to conventional materials. At the same time, specimens with a higher proportion of fibers can be compared with aluminum alloys.

## 2. Multirotor UAV System Description

Multirotor-type UAVs represent an extremely complex system in terms of design and control. Such a type of aircraft is an inherently unstable system, which results from the fact that it cannot independently return to the point of balance (hover) if it loses the functionality of the control loops but will fall or begin to move uncontrollably in space. Furthermore, multirotor UAVs are nonlinear systems since rotor aerodynamic forces and moment characteristics are nonlinear functions with respect to angular velocities. The multirotor UAV is mathematically described by a rigid body dynamic model with 6 second-order differential equations, 12 state variables, and *N* input variables, which makes them a multivariable system. From the design point of view, it is important to emphasize that such UAVs are high energy-consumption systems, considering that for the needs of motion, rotary wings are used, which with their aerodynamic forces, among other effects, must cancel the gravity force.

**Figure 1:**
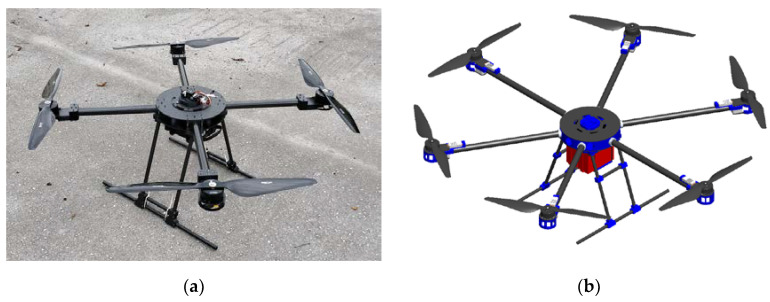
Multirotor UAV platform: (**a**) quadrotor heavy-lift prototype; (**b**) hexarotor 3D model.

The number of control variables depends on the aircraft configuration, which is determined by the geometric arrangement of the rotors. Conventional configurations are characterized by a planar arrangement of rotors. Typical designs are configured with four rotors, the quadrotor or quadcopter [29], with six rotors, a hexarotor, and with eight rotors (octorotor). Typical configurations are shown in Figure 1, the first heavy-lift quadrotor prototype and the 3D model of the hexarotor assembly that was presented within the modular configuration in the previous research [30].

Multirotor UAV dynamics are influenced by the forces and moments of the environment and of the propulsion subsystem. The equations of motion can be derived using the Newton–Euler method. The gravitational force, external disturbances, such as wind gusts, air resistance, gyroscopic effect, and others, act on the rigid body of the multirotor UAV. The only vector through which the dynamics of the aircraft can be directly influenced is the control vector, containing forces and moments of the propulsion configuration (subsystem, module). The multirotor UAV propulsion configuration is defined by the geometric arrangement and characteristics of the rotor. The configuration geometric arrangement is defined by the position and orientation of each rotor in relation to the aircraft coordinate system, and a more detailed derivation is presented in the previous research [9,28]. In conventional propulsion configurations, the rotors are electric propulsion units (EPUs) whose central part is the brushless DC (BLDC) motor driven by an electronic speed controller (ESC). This type of electric propulsion converts electrical energy obtained from lithium-polymer (LiPo) batteries into mechanical work. Fixed-pitch propellers mounted on the rotor axis of the BLDC motor, with their rotation, create aerodynamic effects, from which it follows that the aircraft dynamics directly depend on the rotors’ angular velocities.

When it comes to the design of specialized multirotor UAVs, such aircraft are produced in small series and often exist as unique systems designed to perform specific tasks. In small series production, the price per unit increases dramatically; therefore, it is important to consider technologies for rapid prototyping where manufacturing costs are not sensitive to changes in production volume. In general, it can be said that the design of the aircraft system primarily depends on the purpose, that is, the profile of the mission that the aircraft typically needs to perform. The aircraft system can be divided into subsystems, where from the aspect of designing and mass budgeting, each subsystem is defined by its mass. Payload (equipment and cargo) is determined by the UAV purpose and further dictates the choice of parameters and components of other subsystems. The total mass of the UAV obtained by adding the masses of the subsystems represents the take-off mass of the aircraft.

The basic performance of the multirotor UAV is defined by the ratio of the maximum thrust of the propulsion subsystem in the vertical aircraft axis and the take-off mass. The unwritten rule is that this thrust-to-mass ratio (TMR) is approximately two, except in extreme situations such as racing drones, and a more precise ratio can commonly be read from the specifications of the propulsion components manufacturers. As mentioned earlier, multirotor UAVs are characterized by high energy consumption, which in turn depends on the aircraft’s mass. When designing a system, the ratio of battery mass and capacity is one of the key data. The propulsion and energy subsystems are mutually dependent because, for example, as the power of the aircraft increases, so does the need for energy, which results in a larger mass of the aircraft. The design of the propulsion subsystem is the most complex part of the overall design in terms of the mechanical properties that the parts of the assembly should possess. The multirotor type of UAV can be used in a wide power range, from several tens of watts to several tens of kilowatts, so it is necessary to choose materials and technologies concerning the selected propulsion components. In the aircraft design phase, the parts of the frame that connect everything into a functional assembly have to be modeled and manufactured based on the selected components, where the main requirements are high strength and absorption energy, and low specific mass (weight).

The production of parts for specialized multirotor UAVs can be divided into two main phases. The first phase represents the phase of designing the aircraft where various CAx techniques and tools are utilized, which are outlined through previous research [31,32,33]. In this phase, parts and assemblies are modeled using a 3D software package, and also simulations of airframe parts that need to meet certain mechanical properties can be carried out. The final versions of the 3D CAD model are exported performing a triangulation process to STL format, which is further used for the production of parts. In this research, the SOLIDWORKS software package (Dassault Systèmes SE, Vélizy-Villacoublay, France) is used for 3D modeling purposes. The next phase is the production, i.e., prototyping of the airframe parts. The first step of the prototyping phase is setting print parameters in accordance with the selected AM technology. The print parameters are adjusted in the software package, the slicer, which makes up the software package of a particular 3D printer. After setting the parameters, the G-code is generated for the 3D-printing execution, with which the 3D printer creates a physical model. The last step in this phase is the post-processing of the parts, such as removing the support, or sandblasting and cleaning the parts produced by the SLS technology.

**Figure 2:**
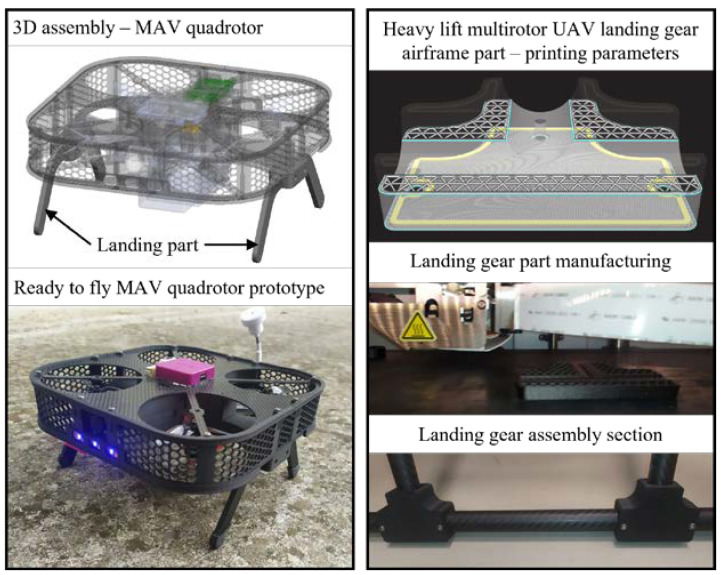
Examples of 3D-printed airframe parts.

Typically for a multirotor UAV, it can be stated that the mechanically loaded parts of the platform can be divided into propulsion airframe parts and landing gear. Whether it is the forces and moments of the propulsion units that act on airframe parts or the forces during landing that occur on the landing gear assembly, such parts are so far developed and tested using CFF technology. For a small UAV that can be classified as a micro aerial vehicle (MAV), landing airframe printed parts are depicted in Figure 2, alongside the heavy-lift multirotor UAV landing gear section. Heavy-lift multirotor UAV landing gear assembly consists of printed parts and prefabricated composite elements, such as tubes, and the whole assembly is shown in Figure 1a.

## 3. Additive Manufacturing Considerations and Experimental Testing

Given that multirotor UAVs are used in various applications where small series or prototypes are used, it is important to consider rapid prototyping technologies in the system design phase. This is an additional benefit from the aspect of using such aircraft in engineering education and research. In addition to the fact that small series rapid prototyping technologies are cheaper compared to conventional technologies, they also significantly reduce development time through rapid iteration and the possibility of early and frequent testing of many different designs or partial designs with critical features. The development of AM and new materials has led to an increase in the quality of the product; it is used more and more often in various industries, such as biomedicine, the automotive industry, and the space industry [34]. AM technology enables the production of structural parts with the required accuracy and mechanical properties at increased production speed, given that the development procedure is relatively simple for different technologies.

### 3.1. Considered Additive Manufacturing Technologies

In the field of making UAVs that are used mainly for research or education, FDM 3D-printing technology is mainly utilized, and recently, due to the availability of cheaper equipment and materials, SLS technology is prominent. In this paper, specimens made with SLS technology using the Lisa 3D printer by Sinterit (Sinterit sp. z o.o., Kraków, Poland), and using Sinterit Studio software (1.7.0.2), were investigated. For the considered FDM technology, a series of specimens can be made on a low-cost Prusa i3 MK3s 3D printer (Prusa Research a.s., Prague, Czech Republic) using the corresponding Prusa Slicer software (2.4.0). For UAV airframe parts that are mechanically loaded, composite materials are used, usually carbon fiber tubes and plates, from which structural parts can be quickly made. This means that it takes relatively little time from the design phase to the production of the aircraft, which is why it is cheaper. Although 3D printing is widely used, it is mostly for smaller UAVs. In the case of, for example, heavy-lift UAV, it is necessary to design parts that are mechanically more loaded, and most often, the propulsion airframe parts are made by processing aluminum. The development of CFF 3D-printing technology enabled the production of parts made of polymer-reinforced materials whose mechanical properties can be compared to aluminum parts, especially in combination with composite parts. In this work, the emphasis is on testing the specimens produced by CFF technology, where the Onyx Pro 3D printer (Markforged, Waltham, MA, USA) by Markforged is utilized.

**Figure 3:**
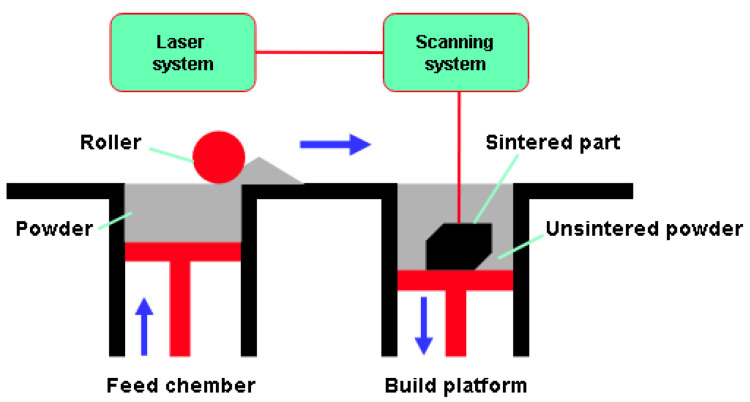
SLS technology—schematic overview.

Regardless of the type of AM technology, the production of the part is based on layer-by-layer construction, which is also the case with SLS technology. This AM technology uses powder materials that are sintered by thermal energy generated by a laser. Modeling by this process can be applied to all materials with powder particles that are sintered due to the application of heat. The most commonly used are polymer powder materials, the most important of which are polycarbonate (PC) and polyamide (PA). To further improve the mechanical and thermal properties of the material, reinforced polymers can be used, where fiber reinforcement is added to PA materials. Unlike other considered technologies in this work, SLS technology is suitable to produce small series of parts with complex geometry. A schematic representation of the working principle is shown in Figure 3, where the manufacturing process begins with the creation of a laser beam that is directed by the laser system to the exact desired position on the XY plane of the printed part. Changing the height of the layer is most often performed by lowering the build platform, the powdered material needed to create a new layer is added from the material feed chamber via a roller. The process of making the layer starts as a result of the action of the laser beam into the powder material. The required temperature applied to the powder particle must be between the crystallization temperature and the melting temperature of the particle [35]. In this research, test specimens were made from polyamide material with the trade name PA12 [36], using Sinterit Lisa Pro 3D printer Sinterit Studio software.

**Figure 4:**
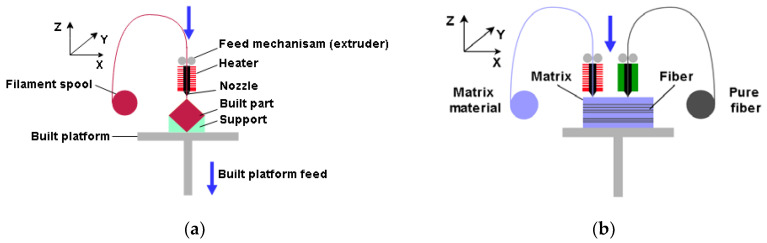
AM technologies—schematic overview: (**a**) FDM; (**b**) CFF.

FDM 3D-printing technology is the most popular and most frequently used technology in research and development, education, and industry. It is based on the melting of solid polymer materials into a semiliquid that passes through a nozzle, forming objects by applying the polymer layer by layer. The working principle of FDM technology is schematically represented in Figure 4a. The created objects are made of thin layers of material whose direction of application defines the mechanical properties of the anisotropic material. Compared to other AM technologies, FDM has its advantages, such as the variety of hardware/software and low material costs, which is why it is the most widely used. Since it is applied layer by layer, the support material is printed for parts with overhanging geometries [37]. Several issues need to be addressed related to mechanical properties, dimensional accuracy, consistency, and undetected defects within the structure of the products. The most used thermoplastic materials for FDM technology are ABS, PLA, and PETG. In this work, test specimens were made from PLA [38] and PETG [39] filament.

Due to the FDM working principle, by adapting this technology it is possible to produce parts that are reinforced with composites. Considering the type of composite with which the polymer material is reinforced, they can be divided into fiber reinforcements, particle reinforcements, and nanoparticle reinforcements. Composite materials are usually synthetic carbon fiber (CF), glass fiber (GF), and Kevlar fiber. Additionally, more environmentally friendly natural biodegradable fibers can be produced [40]. Polymer materials with a reinforced matrix, have significantly increased strength and are used more and more, considering that at the same time they have a low mass [41]. In this paper, the CFF technology with direct reinforcement is discussed, which requires two nozzles, one for the matrix material and the other for the reinforcement fibers, where a composite sandwich structure is formed, as schematically shown in Figure 4b. The system with two nozzles can produce parts only from matrix materials (FDM), and the paper will examine a series of specimens produced from micro carbon fiber-filled nylon material, commercial name Onyx [42], using Markforged equipment and a slicer.

Utilizing the Onyx Pro 3D printer, this system of separate nozzles is used to produce parts of the sandwich structure of the composite, where the Onyx material is used as the matrix and the fiberglass as reinforcement. The mechanical properties of the parts depend on parameters that can be adjusted, such as the number of reinforcement layers and the different geometric arrangements of fibers. Models made with CFF technology have an advantage over those made with conventional FDM technology in terms of significantly higher tensile strength, depending on the proportion of fibers in the composite structure. Test specimens with a different number of reinforcement layers and with various fiber orientations were considered in the testing phase.

### 3.2. Experimental Methods and Equipment

**Figure 5:**
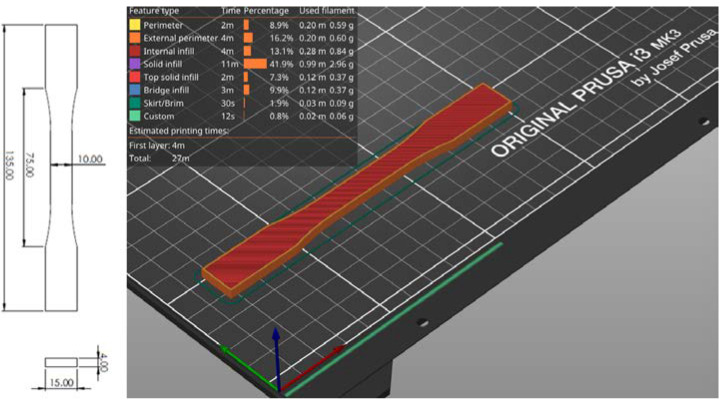
Test specimen (ISO 527-2 [45] standard test specimen for uniaxial quasi-static tensile testing)—G-code generation in a slicer.

**Figure 6:**
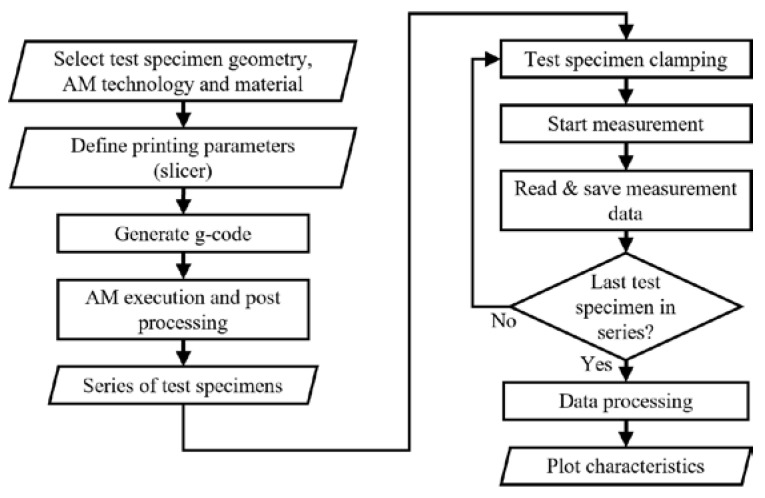
Additive manufacturing and experimental measurements flow chart.

Numerous methods have been used so far to test the mechanical properties of printed materials, such as the Charpy test [43] or rotating bending fatigue analysis [44]; in this work, uniaxial tensile testing is utilized. Test specimens according to the ISO 527-2 standard (Figure 5), with a square cross-section, were modeled in the SOLIDWORKS software package, then exported in .stl format suitable for 3D printing. The print orientation of the test specimens placed on the XY plane of the 3D printer is shown in Figure 6. Researching the literature has determined that the print orientation where the direction of the layers is perpendicular to the direction of the test force gives the lowest values of tensile strength due to simple delamination of the layers. For this reason, such an orientation of the test specimens will not be examined. The data described by the 3D CAD model, according to which the test specimen is made, is inserted into the software called slicer, which is the link between the real and digital models.

Figure 6 shows the basic flowchart of the conducted experiments for one series of test specimens. In the first part, the process related to additive manufacturing is presented, where, regarding the experiment, specimen geometry is selected. Depending on the AM technology, in slicer software, printing parameters are defined. Steps and movements that the printer will perform during the creation of the physical part are described by G-code. The generated G-code is transferred to the printers where the manufacturing begins. It is possible to print one test specimen at a time, or it is possible to print the whole series at once, regardless of the AM technology. When printing test specimens with FDM and CFF technology, there is no post-processing because, due to the geometry of the test specimens, there are no support structures. With SLS technology, it is necessary to clean the test specimens of powder using sandblaster hardware. After the test specimens have been produced for one series, the second part of the process related to the implementation of experimental testing, data processing, and display begins. The first step in this part is the preparation of a test specimen in the clamp of the experimental hardware. After the specimen is clamped, the measurement can be started for the selected test parameters. During the test, data acquisition is performed, and raw data are obtained. Depending on the used equipment and software, in addition, experimental software can generate a report with a graphic display of the test results, as was the case in this research. After the last specimen in the series is tested, the raw data are further processed and interpreted. In this research, the MATLAB software package is used, and the results are graphically presented in the form of stress–strain diagrams.

**Figure 7:**
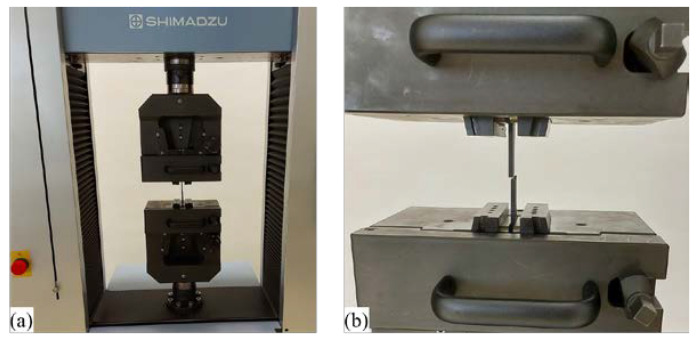
Experimental equipment: (**a**) SHIMADZU AG-X; (**b**) performing quasi-static uniaxial tensile stress on the test specimen.

The test specimens were subjected to a quasi-static uniaxial tensile test on a Shimadzu AG-X plus universal industrial equipment (Figure 7), which can achieve a tensile test force of up to 100 kN. The test was carried out at a constant speed of 30 mm/min without the use of external stress reading devices. The accompanying software of the equipment creates a stress–strain diagram from which the mechanical properties of the material and the critical points of the stress–strain diagram can be analyzed. Raw data measured over a certain time interval during the test are suitable for interpretation in the MS Excel or MATLAB software package. During the testing of polymer materials, clamping problems occur. Due to the lower tensile and compressive strength of the specimen, it is not possible to clamp the specimens into packs with the same force as aluminum and steel specimens because, during the test, the specimen breaks in a place that is not intended for it. These problems are especially pronounced when clamping composite test specimens because the highest values of tensile and compressive strength are expressed in the direction of the fibers, so such values should be achieved in the direction of the test axis, which is perpendicular to the forces that act when clamping the specimens. For this reason, by increasing the tensile strength of the composite specimen, a slip can occur between the packs and the clamped specimen.

### 3.3. Selection of Test Specimens Technological Parameters

In addition to striving to achieve the highest possible tensile strength of the tested specimens, it is more important to express the limit of elasticity. Due to the very small differences between the elastic limit and the maximum tensile strength of the printed specimens, the maximum tensile strength is used as an orientation value for testing purposes. The construction of unmanned aerial vehicles must ensure sufficient tensile strength and criteria of rigidity and stability. In order for the aircraft to be in a functional state, its structural parts must remain in the elastic region; that is, there must be no permanent deformations in the plastic region that would cause displacements of the structure that could adversely affect flight dynamics. The forces that occur due to thrust during flight would deform ductile materials, so it is desirable to have as much rigidity as possible in the construction material. To test the mechanical properties of airframe parts that are statically loaded with the load carried by aircraft, the best description is provided by the uniaxial static tensile test that was carried out in this research. The criteria that the material must meet are low mass, high stiffness, higher elastic limit, and orientation of composite fibers that will be satisfactory in different directions of force.

For this reason, a uniaxial quasi-static tensile test is performed on standard test specimens, from which a stress–strain diagram is obtained. During production, layer by layer, the anisotropic property of the material occurs. Due to the dimensional inaccuracy of the production, non-homogeneity also occurs, which results in different mechanical properties of the material and is present in all printing technologies, unlike mold-injected parts. This is why it is necessary to test several AM technologies using different materials and 3D-printing parameters. Five test specimens are produced for each specimen series (S01–S12). Low-cost PLA and PETG materials were considered, from which specimens (S01–S06) are produced by FDM technology using Prusa equipment and a slicer. Standard printing parameters were investigated, and furthermore, the infill percentage and the number of edge layers were varied. Table 1 shows the materials and parameters of 3D printing for test specimens manufactured using FDM technology. The test specimens (S01–S06) have four floor layers, six roof layers, and a triangles infill pattern. To make a test specimen (S07) with SLS technology, the factory parameters set in Sinterit Studio software are used, and the test specimens are composed of PA12 material.

The stiffness of the non-composite polymer materials used in the tests made by FDM and SLS technology is sufficient for the production of parts of UAVs, while their strength is low for the production of functional, structural airframe parts such as parts of the UAV propulsion assembly. The goal is to achieve the highest possible strength of the test specimens compared to conventional FDM and SLS technologies and to be comparable to aluminum alloys. Due to the most similar conditions when UAVs hit the ground or other unpredictable objects, a car chassis is compared as a reference, where it is tried to achieve a yield strength equal to or greater than the aluminum alloys used for the production of structural car chassis. This is made possible by CFF 3D-printing technology, where composite specimens are obtained by combining matrix and reinforcement materials. Then, micro carbon fiber-filled nylon matrix material is investigated, from which test specimens (S08) with default (factory) parameters (triangular infill 37%) are produced by a Markforged Onyx 3D printer. Reinforcement materials are made from different fiber orientations with the aim of achieving the required values for the design of UAVs. The considered 3D-printing parameters of composite materials are shown in Table 2.

**Figure 8:**
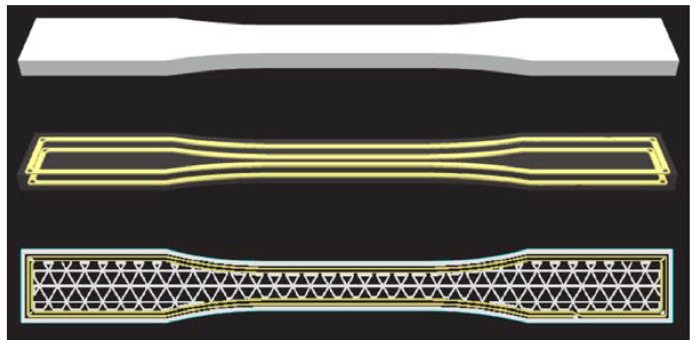
S09 test specimen with concentric fiber reinforcement.

The mechanical properties depend on the number of reinforcement layers, as well as the reinforcement fill type. Changes in mechanical properties with a change in the proportion of fibers in printed composites with fiberglass reinforcement are noted in the paper [46]. The initial specimen consisted of four layers of fiberglass with a total volume share of approximately 4%, so for subsequent specimens, 4 layers were added up to 30 layers of fiberglass (33% volume share). They did not reach the limit of 40% to 50% fiber content in CFF; however, it was clearly proven that with each increase in fiber content, the maximum tensile strength of the tested specimen increases. The arrangement of fibers can be divided into concentric, which is shown in Figure 8, and isotropic. The concentric fiber pattern orientation has fibers in the directions as the walls in the FDM technology following the wall of the model, and for that pattern, a series (S09) of test specimens were 3D printed according to standard Markforged parameters. Isotropic fiber orientation represents parallel fiber lines at different angles. Other test specimen series with different numbers and fiber orientations were made using this pattern approach.

**Figure 9:**
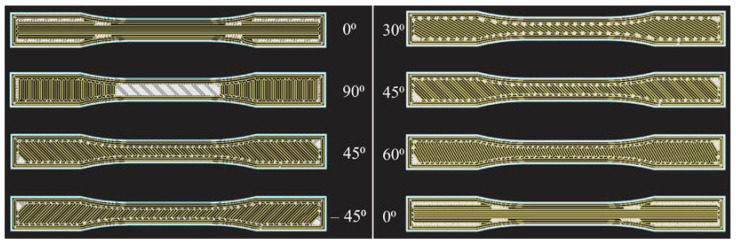
CFF—fiber reinforcement angles.

Fibers in the axial direction have very high strength, but also composites with the action of force perpendicular to the direction of the fiber have the lowest strength, which is why it is necessary to make a compromise with the orientation of the fibers because, in reality, there are multiaxial forces acting on the structure. Placing the fibers longitudinally at 0 degrees in the load direction gives the highest tensile strength. By rotating the fibers (increasing the angles), the tensile strength of the composite material decreases because the applied force is no longer only longitudinal to the fibers but also to the layers of the matrix. By increasing the angles from 0° to 90°, more and more load is transferred to the matrix, which has a significantly lower tensile strength than the fibers, and thus the overall tensile strength of the composite material decreases. In the research [47], such behavior of the material was shown, where the orientation of the fibers was varied by increasing the angle by 22.5° from 0° to 90°. When constructing the airframe of UAVs, the bending load on the rotor arms caused by the thrust of the propeller has a great influence on the airframe construction. When making such parts from composite structures, it is essential to take into account the anisotropic properties of CFF specimens that, due to bending, cause variations in the strain rate, increasing the shear stress between layers that lead to material failure [48]. The behavior of the flexural properties by testing the material is described in the work [49], where increasing the angle of the fibers increases the ductility of the tested specimens. With the structure of the specimens with angles [+45°/−45°], they could not break due to high ductility; however, by adding more layers, a more balanced structure is obtained [48], which is the reason for testing the orientation [30,45,60,0]. The test is carried out for three different setups (S10–S12) of isotropic reinforcement parameters, which are shown in Table 2. Figure 9 shows the geometric arrangement of fibers considered in the test.

### 3.4. Experimental Testing Procedure Integration in Design Process

Technological parameters of test specimens are adjusted for certain technologies in software tools that come with 3D printers. This step is also the first step in the production of the airframe parts that make up the UAV system, and the output of the step is the G-code that is executed on the 3D printer. In general, after adjusting the considered parameters of the 3D model (part or test specimen), it is necessary to prepare the material in the additive manufacturing execution step, which is in the form of filament for FDM and CFF technologies and in powder form for SLS technology. With CFF technology, for the used material, it is necessary to dehumidify the box in which it is placed, and before starting printing, it is necessary to create a purge line of ejected material located between the dry box and the nozzle. With SLS technology, a sieve is used to prepare the material, which is fed into the feed chamber. After the material is ready for production, the G-code execution itself follows. The execution time depends on the technology and the parameters of individual technologies. After the end of printing, depending on the technology, the parts need to be post-processed.

**Figure 10:**
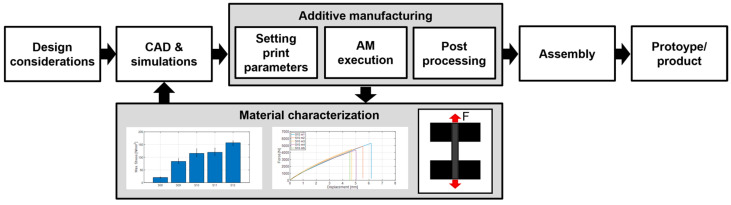
Integrated experimental procedure for material characterization.

When it comes to test specimens, after the production of a particular series is finished, the testing stage is approached. Figure 10 schematically shows the material testing process, the integration of which in the design process enables a more efficient design of structural parts and the aircraft assembly in general. Furthermore, the testing process may include other methods, such as the three-point flexural test or the Charpy impact test. In the next chapter, the test results and characterization for the considered series of specimens (S01–S12) are presented.

## 4. Results of Experimental Testing

Investigation of the mechanical properties of the printed specimens by a uniaxial tensile test is shown by the stress–strain diagrams. For each series of different specimens that are shown in Table 1 and Table 2, five test specimens were used to obtain more reliable results. Each specimen in the series from S01 to S12 is shown separately on the stress–strain diagrams.

### 4.1. Measurement Results Shown by Stress–Strain Diagrams

**Figure 11:**
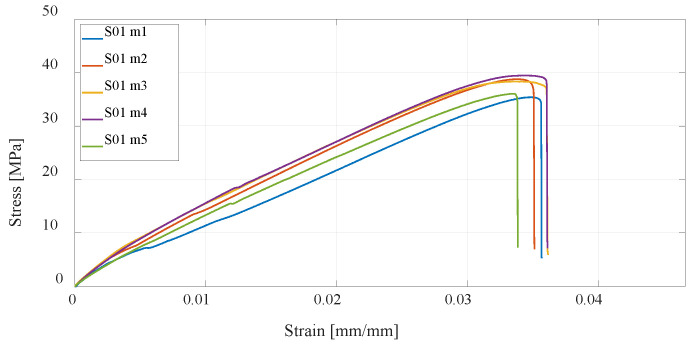
Stress–strain diagram for specimen 1 (S01) series experimental measurements.

**Figure 12:**
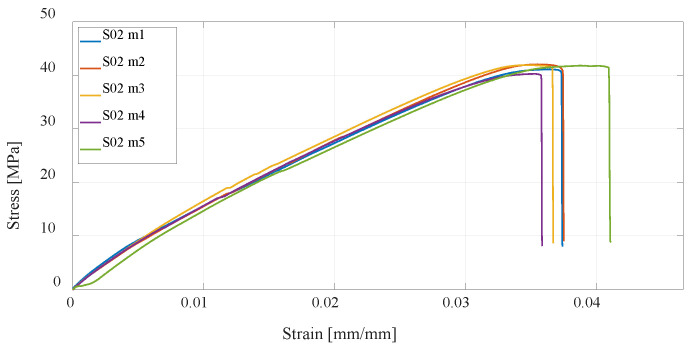
Stress–strain diagram for S02 experimental measurements.

**Figure 13:**
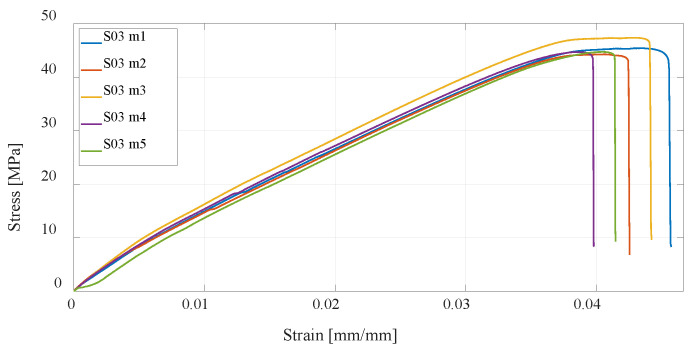
Stress–strain diagram for S03 experimental measurements.

The first group of materials made by the FDM technology consists of specimens S01, S02, and S03, which are composed of PLA material. The mechanical properties depend on the parameters of the print, and this paper considers the infill (percentage) and the number of vertical shells. The first series of specimens use the default settings; in the second series, the percentage of infill has been doubled, while in the third series, the number of vertical shells has been doubled. From the measurement results (Figure 11, Figure 12 and Figure 13), it can be seen that with an increase in the number of vertical shells, the mechanical properties improve more significantly in relation to an increase in the percentage of infill, which can further serve as a milestone in the design of airframe parts that will be made by the FDM technology from PLA material, which require higher tensile strength. Although slightly, S03 specimens are also lighter comparing S02 specimens, and the printing time is slightly shorter.

**Figure 14:**
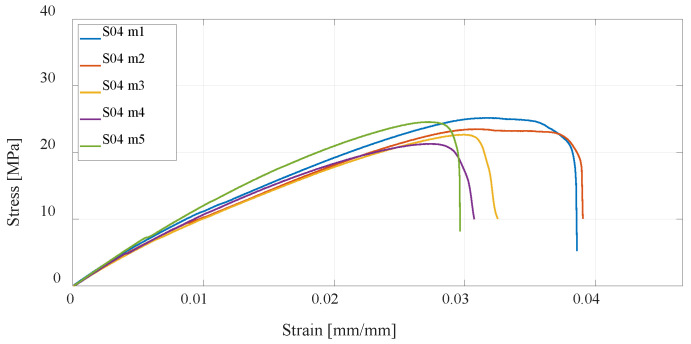
Stress–strain diagram for S04 experimental measurements.

**Figure 15:**
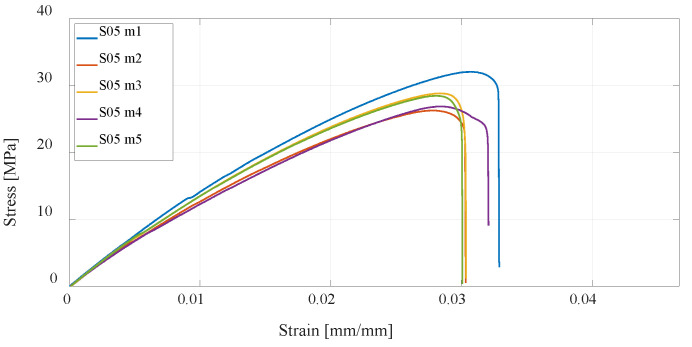
Stress–strain diagram for S05 experimental measurements.

**Figure 16:**
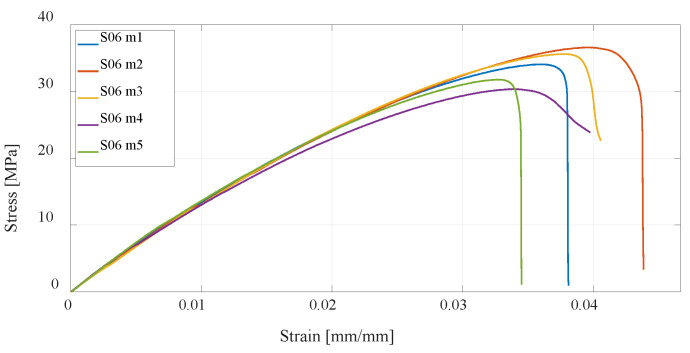
Stress–strain diagram for S06 experimental measurements.

The next three series of test specimens are also produced using the FDM technology but from PETG material. The same 3D-printing parameters were considered, and as with PLA material, it can be concluded that specimens with a higher percentage of infill have higher tensile strength than the default settings, while specimens with twice the number of vertical shells have the highest, as shown in Figure 14, Figure 15 and Figure 16. In relation to PLA material, it is important to emphasize that PETG material generally has lower tensile strength but is more resistant to elevated temperatures and chemical influences.

**Figure 17:**
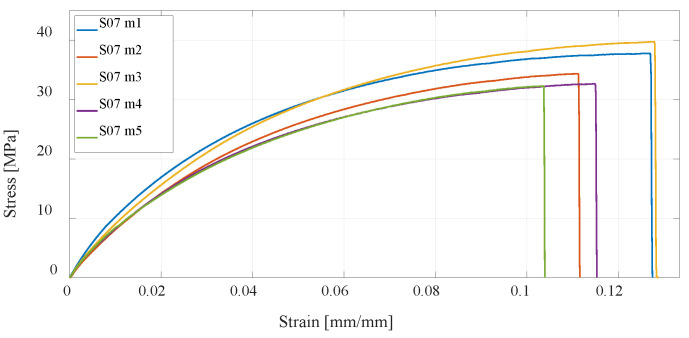
Stress–strain diagram for S07 experimental measurements.

Utilizing the SLS technology, one series of test specimens is manufactured using PA12 powder material. Compared to the test specimens made by the FDM technology from PLA and PETG materials, S07 has significantly higher ductility and higher tensile strength. Due to the manufacturing process, a greater homogeneity of the material is obtained compared to the FDM technologies; thus, there is greater independence of the direction of the force acting on the specimen, which is more credible for real structural UAV airframe parts, which are rarely loaded uniaxially. Figure 17 shows the results of the experimental test for S07 specimens.

**Figure 18:**
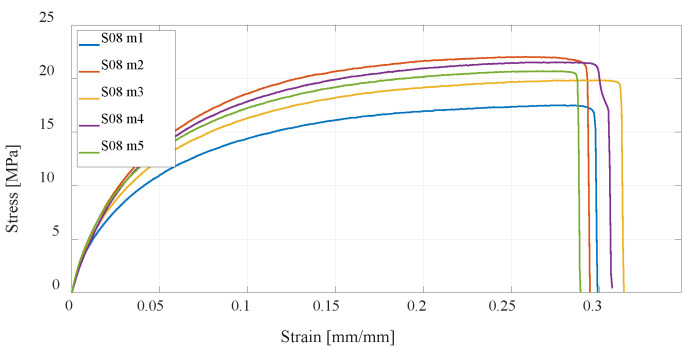
Stress–strain diagram for S08 experimental measurements.

The next series of test specimens will be produced using Markforged equipment and software, which, in addition to the classic FDM technology, also enable the production of parts using the CFF technology. First, a series of specimens made of Onyx carbon fiber-filled nylon material, without reinforcement, is tested. For this material, the highest ductility is obtained, but also the lowest tensile strength (Figure 18). Due to its high ductility, which causes displacement of the test specimen, and tensile strength values close to those of PETG and PLA specimens, a significantly larger area under the curve of the graph is obtained, i.e., absorption energy of the specimen. From the point of view of the impact of the UAV on the objects, it is very favorable; however, larger displacements at lower load forces would lead to problems in flight dynamics. Due to its high ductility, this material is suitable for use as a matrix of a composite structure that is tested further.

**Figure 19:**
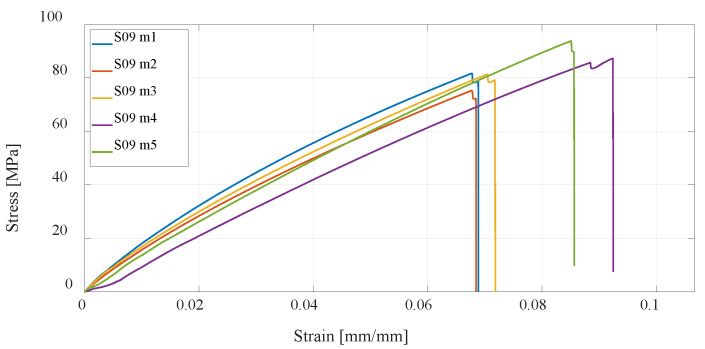
Stress–strain diagram for S09 experimental measurements.

**Figure 20:**
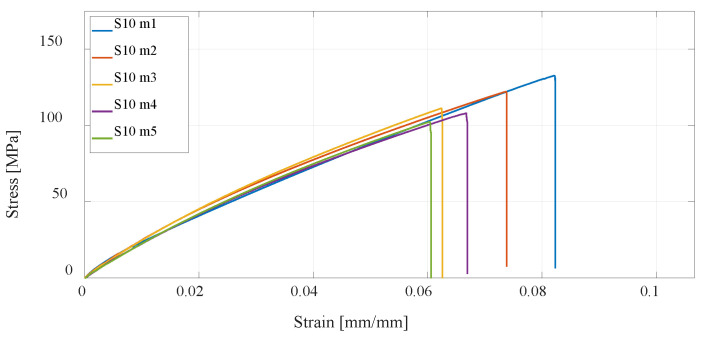
Stress–strain diagram for S10 experimental measurements.

**Figure 21:**
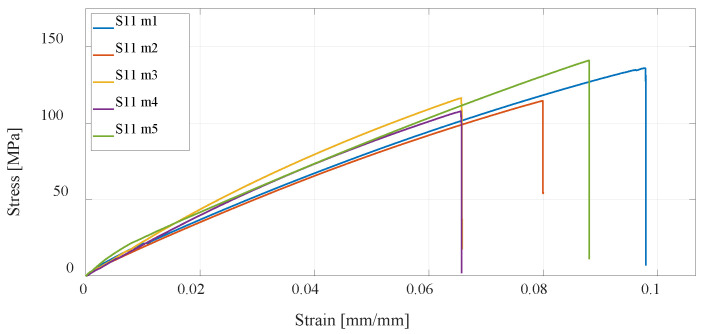
Stress–strain diagram for S11 experimental measurements.

**Figure 22:**
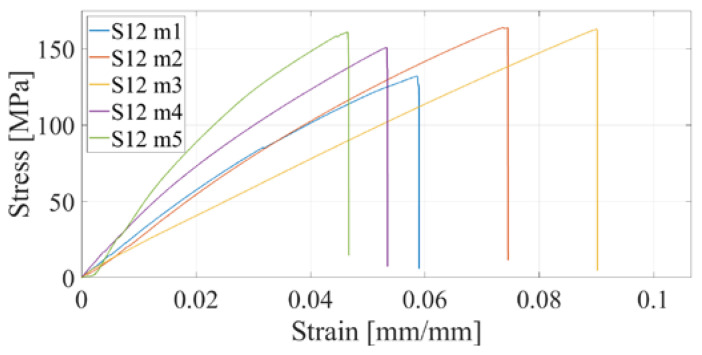
Stress–strain diagram for S12 experimental measurements.

Furthermore, the CFF technology is considered, which enables the production of reinforced composite materials such as parts reinforced with fiberglass. Anisotropy of printed parts is present regardless of how the printing parameters are selected in the slicer; however, in the case of composite structures, the printed polymer forms only a material matrix that is equally printed in all layers of the part. The second part of the composite structure, the fiberglass reinforcement, can be oriented differently to each printing layer. In this research, four series of test specimens were considered. The first composite structure of specimen S09 was made by concentric distribution of fibers in a standard test specimen, and Figure 19 shows the test results. All other specimens of series S10, S11, and S12 have an isotropic fiber arrangement with different fiber orientations, for which the results of the experimental test are shown in Figure 20, Figure 21 and Figure 22. As can be expected, the most significant increase in the value of the maximum tensile strength has the specimens with an increase in the number of fiberglass reinforcement layers.

As previously described, due to the problem of clamping composite materials into packs, increasing it to 16 layers makes clamping even more difficult. The higher the tensile strength of the specimen, the more difficult it is to clamp; that is, by increasing the fibers, the tensile strength significantly increases in the axial direction of the test specimen but not so much in the radial direction of clamping, so the test specimens break due to excessive clamping. If it is not clamped enough, then slippage occurs, so there is a small range to clamp the test specimen so that it does not break and, on the other hand, so that it does not slip. Precisely because of these problems, only one specimen consisting of 16 layers was tested. As expected, that specimen had an even higher tensile strength. For this reason, in future work, testing on a different test device is planned.

### 4.2. Comparison of Maximum Stress for Different AM Technologies

**Figure 23:**
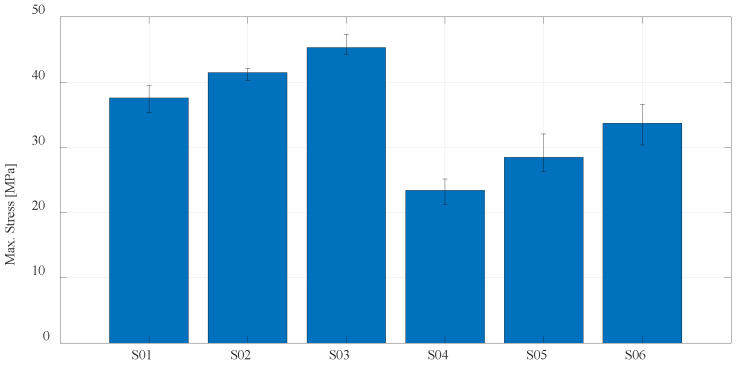
Mean values of the maximum stress regarding PLA and PETG materials.

**Figure 24:**
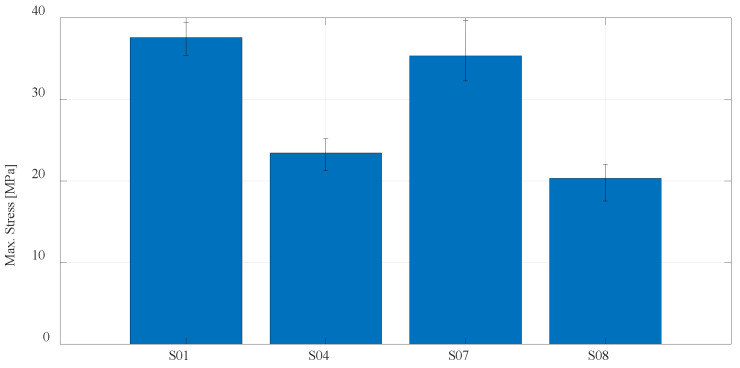
Mean values of the maximum stress regarding default print parameters for PLA, PETG, PA 12, and Onyx materials.

**Figure 25:**
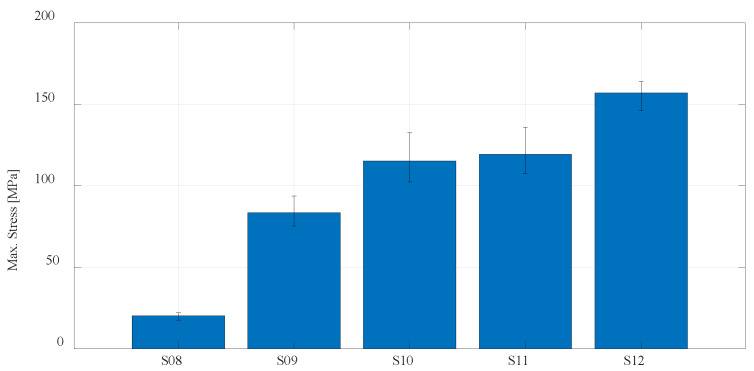
Mean values of the maximum stress regarding CFF technology using Onyx and fiberglass reinforcement.

For a better presentation and visualization of the results, bar charts are used with the mean values of the maximum stress of each series of tested specimens. In the first diagram shown in Figure 23, the values of maximum stress for specimens S01 to S06 made of PLA and PETG materials using FDM technology on the Prusa printer are compared. The diagram shown in Figure 24 shows specimens that actually represent the default parameters for three different technologies and three different printers. Therefore, specimens S01 (PLA) and S04 (PETG) regarding FDM, specimen S07 (PA12) regarding SLS, and finally, S08 (Onyx) regarding CFF/FDM are compared. The following is a comparison of the composite materials that are the main focus of this research. Figure 25 shows all five series of specimens that were produced on the Onyx 3D printer, including the unreinforced specimen S08 to compare with the reinforced specimens.

Onyx material from which specimen series 8 was produced is further used as a matrix material for the production of series S09, S10, S11, and S12. Apart from the fact that the type of reinforcement (concentric or isotropic) and the orientation of the fibers affect the maximum stress, the number of layers in which the fibers are applied has the greatest influence. Considering that of the specimens (S09, S10, S11), which are reinforced with eight layers of fibers, specimen S11 with the highest maximum tensile strength, is further considered, and the proportion of fibers is increased to 12. The specimen S12 with 12 fiber layers orientation is obtained [30/45/60/0]_12,_ which, as expected, gives the highest value of maximum tensile strength. With 12 layers of fibers, the tensile strength is up to 164 MPa. For one measurement that has been completed in the case of 16 fibers, the tensile strength of 174 MPa was recorded.

## 5. Discussion

In this paper, the focus was on CFF technology, which enables the production of multirotor UAV airframe parts more resilient to mechanical loads. For early development and prototyping, it is most advantageous to use FDM technology due to the low cost and faster model creation if mechanical properties are not crucial when prototyping and testing the assemblies and subassemblies. The price of PLA and PETG materials is almost equal and the lowest compared to other technologies, so it is taken as a reference for comparison in terms of costs. Comparing the materials of FDM technology, PLA has a higher tensile strength compared to PETG material for the same printing parameters. The difference between the lowest and highest obtained tensile strength values for the tested materials is not as significant as when changing the technology.

If higher tensile strength and stiffness of the structural airframe part are required, it is more advantageous to use CFF technology because higher values are achieved compared to FDM and SLS technologies. Although different mechanical properties can be obtained by combining the proportion and orientation of the fibers as shown by the experiment, when making the structural part, it is also necessary to consider the manufacturing costs [50]. Onyx material is approximately 10 times more expensive compared to PLA and PETG materials per kilogram. By adding fibers for making composites, this price increases even more, and compared to PLA is approximately 50 times higher. The powder material for SLS technology used in this work is 13 times more expensive than PLA.

Regarding CFF technology, first, tests were carried out on unreinforced specimens (S08), where the lowest values of tensile strength are obtained in relation to all considered AM technologies and materials in this research. However, the highest ductility is obtained for the same material as shown by experiments, which makes this material suitable as a matrix for composite connection applying fiberglass. As possible matrix reinforcements, Kevlar, carbon, and fiberglass can be used. According to the results in paper [51], the maximum tensile strength of the composite consisting of fiberglass reinforcement is higher than Kevlar reinforcement but lower than carbon reinforcement. From the aspect of mechanical properties, carbon fiber reinforcements have better characteristics; however, compared to fiberglass reinforcements, they are between 1.5 and 2 times more expensive. The characterization of test specimens produced by CFF technology with different reinforcement parameters was presented. It is evident from the test results that the considered materials can be compared with aluminum alloys 5xxx and 6xxx, which are used for car frame production, as shown in the paper [52]. In Europe, aluminum alloy 6016-T4 is used for the outer panels of cars, whose yield strength is approximately 110 MPa.

**Figure 26:**
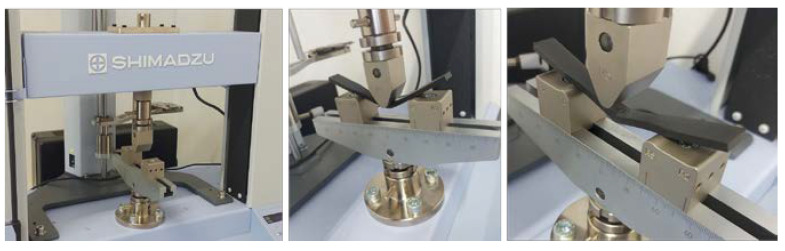
Three-point flexural test—equipment and test execution.

In addition to the various technologies and materials examined in this paper and given that the integration of other experimental setups is considered in future work, preliminary testing of test specimens is conducted using a three-point flexural test [53]. Figure 26 shows the equipment and the execution of the test. Universal testing machine Shimadzu AGS-X 5kN (Shimadzu, Kyoto, Japan) is used, standard ISO 178 [54], with a distance of 62 mm, speed of 1%/min, and preload of 0.5N. Experimental testing of three series of three test specimens was carried out, and the flexural modulus for the tested specimens is presented in Table 3. Based on preliminary testing, a test framework will be established. Moreover, the Charpy impact test will also be integrated into the material characterization process.

## 6. Conclusions

It has been shown by experiments that even by using a concentric pattern of reinforced material, the mechanical properties are significantly improved concerning tensile strength and ductility, which is the goal of obtaining optimal construction of UAVs. The isotropic reinforcement pattern has a higher proportion of fibers; therefore, higher tensile strength and the orientation of the fibers can be modified. A typical way to elevate tensile strength is by increasing the number of reinforcement layers, as shown by the literature and experiments. This results in an increase in the price but also in an increase in production time. In this paper, a contribution to previous research is provided in the form that different fiber orientations were investigated with the aim of increasing the tensile strength for the same proportion of fibers. Three orientations were tested, and the results are presented for two, with the one having the lowest maximum tensile strength of 132 MPa and strain of 6% has S10—[0/90/±45]_8_, which still exceeds the tensile strength properties of some aluminum alloys. The highest value of maximum tensile strength of 136 MPa is obtained for S11—[30/45/60/0]_8_. To achieve higher tensile strength, comparable to the 5xxx and 6xxx series aluminum alloys, an isotropic reinforcement pattern with the highest tensile strength is investigated for the case of 12 and 16 reinforcement layers. For 12 layers, tensile strength is up to 164 MPa, and for 16 layers, one measurement was carried out, and a strength of 174 MPa was recorded, which is comparable to the considered aluminum alloys.

Due to the similar requirements of the construction of UAVs to those of the automotive industry, such as absorption energy, lower mass, and high strength, the composites tested in this paper will be subject to extensive testing, which, in addition to the uniaxial test, includes the three-point flexural test and the Charpy impact test. Moreover, the plan is to analyze the microstructure and the specimens’ fracture. In future work, the goal is to enable a more diverse and extensive comparison with aluminum alloys.

## Figures and Tables

**Table 1:** FDM print parameters.

Specimen Series	Material	Infill %	Wall Layer Number (Vertical Shells)	Mass (g)	Time (min)
S01	PLA	20	2	5.90	27
S02	PLA	40	2	6.62	30
S03	PLA	20	4	6.34	29
S04	PETG	20	2	6.04	27
S05	PETG	40	2	6.78	30
S06	PETG	20	4	6.50	29

**Table 2:** CFF print parameters.

Specimen Series	Material	Number of Reinforcement Layers/Fill Type	Fiber Reinforcement Angles	Mass (g)	Time (min)
S08	Carbon fiber-filled nylon	None	-	6.14	33
S09	Carbon fiber-filled nylon + fiberglass reinforcement	8/Concentric	-	6.24	68
S10	Carbon fiber-filled nylon + fiberglass reinforcement	8/Isotropic	[0/90/±45]_8_	6.95	80
S11	Carbon fiber-filled nylon + fiberglass reinforcement	8/Isotropic	[30/45/60/0]_8_	6.95	82
S12	Carbon fiber-filled nylon + fiberglass reinforcement	12/Isotropic	[30/45/60/0]_12_	7.52	92

**Table 3:** Experimental data for three-point flexural test.

Specimen Series	Flexural Modulus (MPa)			
	Meas. 1	Meas. 2	Meas. 3	Mean
S03	2899	2860	2849	2869
S10	3638	3395	3581	3538
S11	3718	3726	3650	3698

## Data Availability

The data presented in this study can be provided by the corresponding author upon reasonable request.
